# REsiDENT 1 (Re-assessment of Appendicitis Evaluation during laparoscopic appendectomy: Do we End a Non-standardized Treatment approach and habit?): peritoneal irrigation during laparoscopic appendectomy—does the grade of contamination matter? A prospective multicenter resident-based evaluation of a new classification system

**DOI:** 10.1186/s13017-019-0243-4

**Published:** 2019-05-30

**Authors:** Stefano Piero Bernardo Cioffi, Michele Altomare, Andrea Spota, Stefano Granieri, Stefania Cimbanassi, Osvaldo Chiara

**Affiliations:** 1 University of Milan, General Surgery Residency Program., Via Festa del Perdono 7, 20122 Milan, Italy; 2 Niguarda Hospital, Piazza Ospedale Maggiore 3, 20162 Milan, Italy

**Keywords:** Appendicitis, Laparoscopy, Contamination, Lavage, Suction, Intra-abdominal abscess, Infectious complication, Surgical resident

## Abstract

**Background:**

Laparoscopic appendectomy has progressively gained acceptance as the standard of care for acute appendicitis. Focusing on the incidence of postoperative intra-abdominal abscess after a laparoscopic appendectomy, discordant data have been reported ranging from 1.5 to 20%. Besides, evidence advocating advantages from peritoneal irrigation over suction only are lacking. Most studies are burdened by a high level of heterogeneity regarding the severity of the appendicitis and modalities of peritoneal irrigation. One of the main drawbacks is the lack of an accepted classification for different degrees of appendicitis and peritoneal contamination. The aim of the study is to introduce a classification to clarify the relationship between grade of appendicitis, contamination, and postoperative incidence of IAA considering the surgeon’s attitude toward irrigation or suction alone. Preoperative, intra-operative, and postoperative predictive factors for infectious complication will also be assessed. This study is meant to be the first Italian multicenter resident-based observational study.

**Methods:**

Patients suffering from acute appendicitis will be enrolled during a 1-year period, according to inclusion and exclusion criteria. Participants will fill an online form reporting all clinical and intra-operative data of each patient undergoing a laparoscopic appendectomy. General surgery residents will be responsible for data collection. Our proposal of classification is based on the histological grade of appendicitis and intra-operative degree of peritoneal contamination. For each grade, a progressively increasing score is assigned.

**Discussion:**

The observational nature of this study is mandatory to examine surgeons’ attitude toward peritoneal contamination during laparoscopic appendectomy for appendicitis. Identification of different severity grades of acute appendicitis and their relationship with the development of postoperative abscesses is necessary. The resulting classification and score, even considering peritoneal lavage or suction alone, will define risk classes of peri-appendiceal contamination each one related to a specific incidence rate of postoperative IAA. Nowadays, maximum effort should be made to reach the best procedural standardization and surgical decision-making should be supported by solid evidence, especially in an emergency surgery setting.

## Introduction, background, and rationale

Acute appendicitis (AA) is one of the leading causes of acute abdominal pain, and its highest incidence is among children, adolescents, and young adults. The lifetime risk is 8.6% and 6.7% respectively for males and females. Considering the progression of the disease, the therapeutic mainstay is surgical removal of the appendix [1, 2].

Laparoscopic appendectomy has gained acceptance as the standard of care for acute appendicitis after several randomized controlled trials (RCTs) and case-control studies demonstrated clear advantages. Compared to an open appendectomy (OA), a laparoscopic appendectomy (LA) has better outcomes in terms of surgical site infections, time to oral intake, and length of stay (LOS). On the other hand, longer operative times are associated with LA. Considering the incidence of postoperative intra-abdominal abscess (IAA), controversies have been reported during the last few years. A trend toward a higher incidence of IAAs was reported in a systematic review in 2010 [3], though no consistent results were seen from more recent papers [2, 4, 5].

IAA is considered an intra-abdominal organ/space surgical site infection as defined in the Center for Disease Control Surgical Site Infection Criteria, introduced in 1992 [6].

Briefly, surgical site infections (SSIs) are divided into three categories: superficial incisional SSI (SI SSI), deep incisional SSI (DI SSI), and organ/space surgical site infection (O/S SSI). Each category has defined inclusion criteria.

O/S SSI must meet the following criteria:

- Date of event within 30 days after surgical procedure
- Plus involvement of any part of the body deeper than the fascial/muscle layers that are opened or manipulated during the operative procedure
- Plus at least one of the following:
  - Purulent drainage from a drain that is placed into the organ/space (for example, closed suction drainage system, open drain, T-tube drain, CT-guided drainage)
  - Organism(s) identified from fluid or tissue in the organ/space by a culture- or non-culture-based microbiologic testing method which is performed for purposes of clinical diagnosis or treatment
  - An abscess or other evidence of infection involving the organ/space that is detected on the gross anatomical or histopathologic exam, or imaging test evidence suggestive of infection
- Plus at least one criterion for a specific organ/space infection site listed in the CDC/NHSN Surveillance Definitions for Specific Types of Infections [7] part of 2019 Patient Safety Component Manual from Center for Disease Control and Prevention and National Healthcare Safety Network (NHSN) [8]

The average reported incidence of IAA following a laparoscopic appendectomy in the literature ranges from 1.5 to 20% with extreme heterogeneity in the types of studies considered [4, 5, 9–11].

Few studies analyzed risk factors for the development of IAA after LA. Body mass index, leukocytosis, perforated appendicitis, and operative time > 90 min were associated with the development of postoperative IAA using multivariate analysis [12–14]. A retrospective single-center analysis performed by Cho et al. with 1822 patients identified intra-abdominal irrigation as the only risk factor associated with IAA [9].

Evidence regarding advantages from peritoneal irrigation over suction only are scarce. Both guidelines from the World Society of Emergency Surgery (WSES) and from European Association for Endoscopic Surgery do not report any advantages from this practice, supported by low levels of evidence (LOE 2, GOR B) [1, 2].

Furthermore, three randomized control trials and two retrospective observational studies have been analyzed in a recent systematic review and meta-analysis. These studies are burdened by a high level of heterogeneity regarding the severity of appendicitis and modalities of peritoneal irrigation [15]. No advantages from peritoneal irrigation have been identified to date. This data are confirmed by a more recent meta-analysis in which heterogeneity of patients has been highlighted [16].

With the current literature, one of the main bias affecting all studies is the lack of a universally accepted classification method for different degrees of appendicitis and peritoneal contamination. Therefore, to establish the effective relationship between contamination, degree of intra-operative irrigation, and postoperative IAA incidence, it is mandatory to apply a specific classification, based on the intra-operative scenario. The grading system already published by WSES for acute appendicitis is a useful tool for a comprehensive evaluation of patients [17]. A system based on an exclusive and accurate focus on peritoneal contamination plus the histological grade of inflammation is nevertheless lacking.

This study is meant to be the first Italian multicenter resident-based study. This is due to the scarce involvement of General Surgery Italian residents in scientific projects. Residents have the responsibility to take care of patient selection, personal involvement in surgery, intra-operative data collection, postoperative follow-up, and related data collection, data analysis, and study writing.

## Hypothesis, design of the study, and aims

The driving hypothesis of our study is that there should exist a relationship between different intra-operative treatment protocols (irrigation vs suction alone) and postoperative incidence of IAA. Furthermore, considering various grades of appendicitis and peritoneal contamination, the use of irrigation vs suction alone could lead to different rates of postoperative IAA.

The aim of this study is to introduce and evaluate a classification for acute appendicitis taking into account the peritoneal contamination to delineate the relationship between grade of appendicitis and postoperative incidence of IAA considering the surgeon’s attitude toward irrigation or suction alone. Some studies described that the incidence of postoperative IAA is higher in the perforated appendix [12, 13]. No clear evidence exists exploring the severity of peritonitis as a potential predictor of IAA and its relationship with irrigation or suction alone.

This is a prospective observational multicenter study.

Primary endpoint:

- Relationship among each severity grade of acute appendicitis and postoperative incidence of intra-abdominal abscesses

Secondary endpoint:

- Impact of intraperitoneal irrigation or suction alone on the incidence of IAA for each severity grade

## Methods

Patients are selected according to the following criteria:

- Inclusion criteria:
  - Patients between 18 and 69 years old
  - Surgical laparoscopic approach for AA
  - Intra-operative and histological diagnosis of AA
- Exclusion criteria:
  - Patients < 18 years old or > 69 years old
  - Previous appendectomy
  - Previous appendicitis treated conservatively
  - Open approach for surgery or intra-operative conversion
  - Co-existence of other intra-abdominal infections (IAI)
  - Patients with immunodeficiency
  - Patients treated with steroid, immunosuppressant, or CHT within previous 6 months

Clinical data:

- Patient ID
- Demographic data (age, sex, BMI)
- Comorbidity (American Society of Anesthesiology classification, Charlson comorbidity index [18])
- Organ failure at admission (predisposition infection/injury response organ dysfunction—PIRO score [19])
- Alvarado score and CRP

Operative data:

- Lavage and suction vs suction alone
- Lavage volume
- Drainage
- Operative time
- Operator: resident (under senior surgeon supervision) vs senior surgeon
- Individual difficulty grading scale of the procedure

Intra-operative findings:

- Grade of appendicitis according to classification (see below)
- Corresponding histology grade
- Presence of single or multiple abscesses
- Presence of localized or diffuse, purulent, or fecal peritonitis

Postoperative data and follow-up:

- Overall length of stay
- Length of stay in ICU
- Antibiotic therapy
- Duration of antibiotic therapy
- Superficial surgical site infection
- Deep surgical site infection
- Surgical site infection organ/space infection
- Single intra-abdominal collection
- Multiple intra-abdominal collections
- Peritonitis
- Clavien-Dindo Classification of surgical complications [20]
- New access in ED with diagnosis of IAA into 30–60–90 days
- Length of stay for new hospitalization

### Classification

Our proposal of classification (Table 1) is based on both the histological grade [21] of appendicitis and intra-operative degree of peritoneal contamination, considering data collected from the literature.

**Table 1:** Proposal of classification

Appendix aspect	
Erythematous and edematous appendix^a^	
Appendiceal phlegmon^b^	
Gangrenous appendix^c^	
Perforated appendix^d^	
Contamination	
Single abscess	
Multiple abscesses	
Localized purulent peritonitis	
Diffuse purulent peritonitis	
Localized fecal peritonitis	
Diffuse fecal peritonitis	

^a^Neutrophils within mucosa and submucosa with mucosal

^b^Neutrophilic infiltration of mucosa, submucosa, and muscolaris propria. Transmural inflammation, extensive ulceration, and intramural abscess; vascular thrombosis

^c^Transmural inflammation with areas of necrosis; extensive mucosal ulceration

^d^Transmural inflammation with areas of necrosis; extensive mucosal ulceration plus parietal disruption [17]

One type of appendix aspect will be assigned to each patient, as well as one type of contamination will correspond to a single patient.

### Data collection and statistical analysis

Participants will fill an online form reporting all clinical and intra-operative data of each patient who will undergo a laparoscopic appendectomy. All personal information of patients will be removed according to data anonymization.

Data will be reported in accordance with Strengthening the Reporting of Observational studies in Epidemiology guidelines (STROBE) for observational studies [22].

Residents from each involved center will be responsible for data collection.

All forms will be collected in an electronic database by the coordinating residents who will check for data completeness. All residents taking part in the study will be asked to enter eventual missing data.

The incidence of IAA after laparoscopic appendectomy reported in the literature ranges from 1.5 to 20% [4, 5, 9–11]. Such a wide interval is not negligible when calculating the sample size (*n*) of the study cohort. Furthermore, some important basic considerations need to be taken into account: appendicitis is the most common acute surgical disease so the baseline population is large and cannot be exactly estimated; the incidence of intra-abdominal abscesses has never been systematically investigated in regard to the severity of appendicitis and contamination. All patients included in the study will be classified into different clusters considering the grade of appendicitis and contamination, lavage vs suction alone, and postoperative incidence of IAA.

Considering all these aspects, sample size for power calculation can be reasonably estimated given a 95% confidence interval with a *z*-score of 1.96, a 50% standard deviation, and a 0.03 margin of error. This formula does not include the incidence rate of IAA for the aforementioned reasons.

The estimated sample size would be around at least 1067 patients. Data will be collected in a computerized spreadsheet (Microsoft Excel 2016; Microsoft Corporation, Redmond; WA) and analyzed with statistical software (IBM Corp. Released 2017. IBM SPSS Statistics for Windows, version 25.0. Armonk, NY).

*χ*^*2*^ test will be assessed to compare categorical variables, and univariate logistic regression will be performed to provide hazard ratios for individual variables, identifying possible predictors of postoperative complication (IAA above all). All significant (*p* < 0.05) variables at univariate analysis will be included in a multivariate regression model in order to detect independent risk factors for the outcome and to estimate odds ratio and 95% confidence intervals.

A scoring system will be built according to the results of statistical analysis. The weight of each variable will be addressed based on odds ratio values. To evaluate the accuracy of the score, discrimination and calibration of the model will be explored. The former will be assessed by receiver operating characteristic (ROC) curve analysis, whereas the latter will be investigated with the Hosmer-Lemeshow goodness-of-fit test. Based on the results of the ROC curve analysis, we will proceed to identify different severity grades of acute appendicitis each one related to a specific risk of developing delayed IAA. *χ*^*2*^ test will be assessed to compare categorical variables, and univariate logistic regression will be performed to provide hazard ratios for individual variables, identifying possible predictors of postoperative complication (IAA above all). All significant (*p* < 0.05) variables at univariate analysis will be included in a multivariate regression model in order to detect independent risk factors for the outcome and to estimate odds ratio and 95% confidence intervals.

## Discussion

The observational nature of this study is mandatory to examine and analyze surgeon attitude toward peritoneal contamination related to appendicitis during laparoscopic appendectomy. The optimal study design to address the core matter would be a well-structured RCT to assess the real effect of lavage plus suction versus suction alone on postoperative IAA. As above reported in the “Introduction, background, and rationale” section, there is a lack of methodological strictness in the existent randomized trials due to heterogeneous determination of appendicitis severity and peritoneal contamination.

We feel indispensable to start from identifying different grades of appendicitis, related contamination, and their relationship with postoperative abscesses, considering surgeons’ attitude. The core step is to classify each patient and create a score to pick out classes of peri-appendiceal contamination related to different incidence rate of postoperative IAA.

The General Surgery residency program at the University of Milan is based on a large clinical network in which residents are distributed in different hospitals. It is a 5-year program. One hundred thirty residents are involved. The clinical network includes 59 surgical units in 27 hospitals.

This system structure allows great exposure to different surgical scenarios, inside and outside the operating room. The program requires, per year, a minimum of procedures in which the resident has to be involved. From the first to the fifth year, the difficulty of the procedure is increased. To the best of our knowledge, this is one of the most efficient residency programs in Italy because great attention is dedicated to surgical skills improvement. On the other hand, this program may underestimate the scientific needs and educational growth of residents. This study is resident driven as an incitement to a defined organization for the scientific growth of the University of Milan residents.

During the project start-up phase of this study, an official group of residents of the General Surgery program at the University of Milan has been created. The main aim of this group of young doctors is to try to guarantee to each resident, who is interested, the minimal scientific knowledge and ability to critically understand, ideate, and project a scientific study.

## Conclusions

The study proposed within this protocol may have different scientific, operative, and prognostic implications. The classification and the scoring system can be fundamental instruments for general emergency surgeon to identify various grades of appendiceal inflammation and peri-appendiceal contamination related to different incidence of postoperative infectious complications considering the use of irrigation or suction alone.

Thus, these instruments can be helpful to reduce selection biases and select homogenous cohorts of patients. The main scientific target should be planning and conducting well-structured studies to reach maximal external validity.

On the other hand, the biggest practical consequence from the application of the score can be the identification of intra-operative scenarios in which the impact of irrigation or suction alone has been clearly outlined. More awareness of the impact of daily surgical practices can help young and trained surgeons during daily surgical practice.

Surgical decision-making should be supported by solid evidence, especially in an emergency surgery setting. If particular aspects of surgical approaches are not adequately supported by clear data, the emergency surgeon cannot be completely satisfied by relying on common sense alone. Nowadays, maximum effort should be made to reach the best procedural standardization, especially during acute care procedures.

## Abbreviations

AA: Acute appendicitis
DI SSI: Deep incisional surgical site infection
IAA: Intra-abdominal abscess
LA: Laparoscopic appendectomy
O/S SSI: Organ/space surgical site infection
OA: Open appendectomy
RCT: Randomized control trial
SI SSI: Superficial incisional surgical site infection
SSI: Surgical site infection
